# Supplementary material for: SARS-CoV-2 PCR and antibody testing for an entire rural community: methods and feasibility of high-throughput testing procedures
Source: Arch Public Health. 2021 Jul 7;79:125. doi: 10.1186/s13690-021-00647-8 (PMC8261397; doi:10.1186/s13690-021-00647-8)
Supplement: Supplementary file 1 — Appendix 1. [file 13690_2021_647_MOESM1_ESM.pdf]

APPENDIX 1: Daily Staffing Requirements with Infection Prevention Guidance

	Job description	Location	Training required				Participant contact	PPE required						PPE changing
			PPE	Flow	Web Platform	Test Procedure		Surgical mask	Maintain 6ft	Eye protection	Gown/suit	Gloves	Respirator	
**COMMUNITY VOLUNTEERS**														
Traffic controllers (n=4-5)	Help manage flow of cars	Parking lot		X			Minimal	X	X					One mask daily
Community Greeters (n=2)	Welcome, give surgical masks to participants. If no appointment, direct back to website or to onsite registration support.	Parking lot	X	X	X		Minimal	X	X	X				One mask daily<br>*Can store mask in paper bag for reuse during day.*
Interpreters (n=2)	Translate English to Spanish from start to finish.	All	X	X			Moderate	X		X		X		Change gloves, hand sanitizer after every participant. all other
**MEDICAL VOLUNTEERS**														
Triage greeters (n=4)	Using tablet, confirm participants have appointments, assess if any in car have symptoms (direct to symptom lane).		X	X	X		Minimal	X	X	X				One mask daily<br>*Can store mask in paper bag for reuse during day.*
Specimen collectors (n=12)	Briefly explains procedure to participant. First cleans finger, uses lancet to perform finger stick, collect and stores blood. Swabs OP then mid-turbinate, drops capped vial into open bag held open by test assistant.	Test bay	X	X		X	Most contact	X		X	X	X	X	Change gloves, hand sanitizer after ever participant. All others once per shift. Can reuse N95 mask, store in paper bag.
Test assistants (n=12)	Ask participant name/DOB and whether any day-of symptoms and verbally report to admin in tent. Pick up basket with correctly labeled microtainer, lancet, band aid, alcohol swab. Hand items to tester as needed. Then hand tester swab, viral transport media. Hold open biohazard bag for phlebotomist to drop swab specimen into.	Test bay	X	X	X	X	Moderate	X	X	X		X		Change gloves, hand sanitizer after every participant. all other equipment changed once per shift. *Must maintain physical distance, particularly testing procedures.*
Administrative volunteers (n=6)	Find record in database (after hearing from test assistant), record participant symptoms. Locate pre-printed label. Label 2 containers and place 3rd label on requisition form. Record whether participant received tests successfully.	Inside tent		X	X	X	Minimal	X	X					Change gloves, hand sanitizer after every participant. all other equipment changed once per shift.
Runners/lab quality control leads (n=2)	Create "test kits" with labeled microtainer, lancet, alcohol swab, gauze, labeled viral transport media, and swab. Ensure specimens secured safely.	Test bay & inside tent		X			Minimal	X				X		
**LEADERSHIP VOLUNTEERS**														
Tent supervisor (n=4)	Help troubleshoot operations, testing procedures, or field questions from participants or volunteers	Inside tent		X	X	X	Minimal	X						One mask daily<br>*Can store mask in paper bag for reuse during day.*
Medical director (n=1)	Help troubleshoot operations, testing procedures, or field questions from participants or volunteers	Inside tent	X	X	X	X	Minimal	X						One mask daily<br>*Can store mask in paper bag for reuse during day.*
