# Supplementary material for: SARS-CoV-2 PCR and antibody testing for an entire rural community: methods and feasibility of high-throughput testing procedures
Source: Arch Public Health. 2021 Jul 7;79:125. doi: 10.1186/s13690-021-00647-8 (PMC8261397; doi:10.1186/s13690-021-00647-8)
Supplement: Supplementary file 2 — Appendix 2. [file 13690_2021_647_MOESM2_ESM.pdf]

APPENDIX 1: Daily screening of study staff by email (based on UCSF Health's daily screen)

	No	Yes
Have you been diagnosed with COVID-19 in the past 14 days?	If no, you may proceed to volunteer.	If yes, please stay home and notify Bolinas study leadership.
Do you live with someone who has been diagnosed with COVID-19 in the past 14 days?	If no, you may proceed to volunteer.	If yes, please stay home and notify Bolinas study leadership.
Have you had any of the following symptoms in the last 14 days (including the last 24 hours)?<br><br>- Fever over 100F<br>- Sore throat<br>- Difficulty breathing<br>- Unexplained muscle aches<br>- Cough<br>- Loss of sense of smell or taste<br>- Nasal congestion different from pre-existing allergies	If none of the above, you may proceed to volunteer.	If yes to *only* nasal congestion, it is possible you may proceed to volunteer. Please contact study leadership.<br><br>If yes to any other symptoms, please stay home and notify Bolinas study leadership.

Have you been in unprotected contact with someone diagnosed with COVID-19 in the past 14 days?<br><br>(unprotected means without full PPE at work or *close* contact in the community)	If no, you may proceed to volunteer.	If yes, please stay home and notify Bolinas study leadership.
Have you returned from travel outside the US or from NY/NJ/CT in the past 14 days?	If no, you may proceed to volunteer.	If yes *and* symptoms, please stay at home and notify Bolinas study leadership.<br><br>If yes and NO symptoms, you may proceed to volunteer with self-monitoring of symptoms twice daily.
