# Supplementary material for: SARS-CoV-2 PCR and antibody testing for an entire rural community: methods and feasibility of high-throughput testing procedures
Source: Arch Public Health. 2021 Jul 7;79:125. doi: 10.1186/s13690-021-00647-8 (PMC8261397; doi:10.1186/s13690-021-00647-8)
Supplement: Supplementary file 3 — Appendix 3. [file 13690_2021_647_MOESM3_ESM.docx]

**APPENDIX 3: Detailed Testing Procedures**

1. Title: Detailed Serum and Respiratory Sample Collection Procedures
2. Supplies:
   1. Basic Materials
   - Alcohol swabs
   - Cotton swabs or Band-aids
   - Sterile disposable finger-stick lancets
   - Puncture-resistant labeled sharps disposable containers
   - Bio-waste bins and liners
   1. Diagnostic Materials
   - BD microtainer red-cap tubes
   - Nasopharyngeal or oropharyngeal-sized flocked or synethic swabs
   - RNA/DNA shield in vials for swab transport
3. Procedures
4. FINGER PRICK COLLECTION METHODS

In order to obtain quality specimens using this method, participants were instructed to warm hands using car heaters prior to testing for optimal vasodilation. Testers selected the 3rd or 4th digit on a participant’s non-dominant hand for the thinnest tissue. After cleaning the selected finger with alcohol, the lancet was applied firmly with prick at the lateral aspect of fingertip. To obtain a sufficient quantity of blood, we used auto-retractable, 17-gauge lancets blade with 2mm depth.
 Next, the tester supinated the participant’s hand to optimize flow of blood, while ensuring well below level of the heart. Blood was collected directly into microtainers (without use of a capillary tube), with a goal volume of 500 microliters. In order to obtain this volume, testers firmly wiped emerging blood at the fingertip into the microtainer while gently “milking” or massaging the finger from the distal interphalangeal area to prevent early clotting. This procedure often took from 1-5 minutes. If the first prick yielded less than 300 microliters of blood, we suggested repeat collection on a different finger if the participant was amenable.
 After successful collection, the microtainer was capped and placed upright in a box with racks that was kept on the side of the tent designated for participant samples. Participants were handed gauze to apply pressure while PCR collection completed, and generally tolerated the prick well with minimal pain reported.

1. RESPIRATORY SAMPLING METHODS

With regard to collection of viral RNA specimens for PCR, we adhered to standard clinical best practices around oropharyngeal and mid-turbinate collection. Anatomic location of swabbing was guided by available swab size, and we collected oropharyngeal then mid-turbinate specimens (using the same swab) to optimize sensitivity. Particular operational considerations specific to this high through-put model included: 1) performing this collection after finger prick to limit spread of droplets given increased risk of sneezing or coughing, 2) instructing participants to keep their own mask in place, but moving down for oropharyngeal collection and then back over mouth for mid-turbinate collection. Finally, we stored vials upright after collection and in transport to optimize sample integrity.
